# Antibiotics: A Bibliometric Analysis of Top 100 Classics

**DOI:** 10.3390/antibiotics9050219

**Published:** 2020-04-29

**Authors:** Anas Imran Arshad, Paras Ahmad, Mohmed Isaqali Karobari, Jawaad Ahmed Asif, Mohammad Khursheed Alam, Zuliani Mahmood, Normastura Abd Rahman, Noraida Mamat, Mohammad Amjad Kamal

**Affiliations:** 1 Paediatric Dentistry Unit, School of Dental Sciences, Universiti Sains Malaysia, Health Campus, Kubang Kerian 16150, Kelantan, Malaysia; anas.i@live.com (A.I.A.); norraida@usm.my (N.M.); 2 Paedodontics Department, Rashid Latif Dental College, Lahore 54600, Pakistan; 3 Oral Medicine Unit, School of Dental Sciences, Universiti Sains Malaysia, Health Campus, Kubang Kerian 16150, Kelantan, Malaysia; docparas2017@gmail.com; 4 Conservative Unit, School of Dental Sciences, Universiti Sains Malaysia, Health Campus, Kubang Kerian 16150, Kelantan, Malaysia; dr.isaq@gmail.com; 5 Oral and Maxillofacial Surgery Unit, School of Dental Sciences, Universiti Sains Malaysia, Health Campus, Kubang Kerian 16150, Kelantan, Malaysia; doctorjawaad@gmail.com; 6 Orthodontic Department, College of Dentistry, Jouf University, Sakaka 72721, Kingdom of Saudi Arabia; dralam@gmail.com; 7 Dental Public Health Unit, School of Dental Sciences, Universiti Sains Malaysia, Kota Bharu 16150, Malaysia; normastura@usm.my; 8 King Fahd Medical Research Center, King Abdulaziz University, Jeddah 21589, Saudi Arabia; prof.ma.kamal@gmail.com; 9 Enzymoics, 7 Peterlee Place, Hebersham, NSW 2770, Australia; 10 Novel Global Community Educational Foundation, Hebersham, NSW 2770, Australia

**Keywords:** citation classics, top-cited articles, antibiotics, bibliometric analysis, antibacterial, antimicrobials

## Abstract

Citation frequencies represent the most significant contributions in any respective field. This bibliometric analysis aimed to identify and analyze the 100 most-cited publications in the field of antibiotics and to highlight the trends of research in this field. “All databases” of Clarivate Analytics’ Web of Science was used to identify and analyze the 100 publications. The articles were then cross-matched with Scopus and Google Scholar. The frequency of citation ranged from 940 to 11,051 for the Web of Science, 1053 to 10,740 for Scopus, and 1162 to 20,041 for Google Scholar. A total of 513 authors made contributions to the ranked list, and Robert E.W. Hancock contributed in six articles, which made it to the ranked list. Sixty-six scientific contributions originated from the United States of America. Five publications were linked to the University of Manitoba, Canada, that was identified as the educational organization, made the most contributions (*n* = 5). According to the methodological design, 26 of the most cited works were review-type closely followed by 23 expert opinions/perspectives. Eight articles were published in Nature journal, making it the journal with the most scientific contribution in this field. Correlation analysis between the publication age and citation frequency was found statistically significant (*p* = 0.012).

## 1. Introduction

The bibliometric analysis provides a quantitative review of literature in any field of research based on the citation frequency of the conducted research. This type of analysis identifies the countries, organizations, and authors who were affiliated with the most prominent scientific contributions [1,2]. The thrust areas of the past research in a specialty can be identified by analyzing the most-cited work currently, which information can then be used to channel the future research. 

The bibliometric concept of “citation classics” was described by the founder of the Institute for Scientific Information (ISI), Dr Eugene Garfield, in 1977. Its purpose was identification as well as acknowledgment of frequently cited research of authors and their peers that would consequently encourage the respective work and its impact on the specialty [3]. The eligibility of a scientific contribution to be counted as a “classic” depends on the specialty being analyzed. While some analysts believe that 100 or more citations of a publication are sufficient [4,5,6,7], others believe that a publication must be cited more than 400 times to be counted in the list [8]. Leading scientific databases like Web of Science (WoS), Elsevier’s Scopus (ES), and Google Scholar (GS) and influential publishers like BioMed Central, Nature, Wiley, Frontiers, Elsevier, and PLOS are developing and embedding options to perform on-site citation analysis [4,8,9,10,11]. 

Several bibliometric analyses have been conducted in other fields of health sciences, which include the specialty of dentistry [4,8,9,12,13] and medicine [14,15,16,17,18]. However, the “classics” in the field of antibiotics has not been identified. The aim is to identify and analyze the top 100 classics in the specialty of antibiotics to highlight the notable advancements made on this very topic over the recent decades.

## 2. Materials and Methods

### 2.1. Search Strategy

Two independent reviewers (A.I.A) and (P.A) conducted a literature search on 21st March 2020 using ‘All-Databases’ collection of WoS. The search terms were identified after consulting field experts from different institutions, and a final search string was developed and agreed upon unanimously. No language restrictions, publication year range, or methodology selections were applied.

### 2.2. Eligibility Criteria

Titles of the articles published in peer-reviewed journals were selected when either of the following search terms identified: “antibiotics” OR “antibiotic” OR “anti-bacterial” OR “antibacterial” OR “anti-infective” OR “anti-infectious” OR “anti-microbial” OR “antimicrobial”.

Articles having less than 400 citations according to the WoS and ES databases were excluded. Articles published in low or no impact factor journals were not included in the marked list.

### 2.3. Data Extraction and Bibliometric Parameters

A total of 124,122 publications were initially identified using the search string described above. The publications were sorted based on the frequency of citations in a descending manner. The list of top 100 classics was marked based on the citation frequency. The marked list was then cross-matched with GS and ES databases. The marked lists from the A.I.A and P.A was then shared with the field experts, and all authors unanimously agreed upon the final list. Bibliometric parameters for the articles available in “All Databases” were recorded from the WoS database, which includes the title of the article, journal title, citation count, current citation index (CCI) 2019 (total citations received in 2019), publication year, names of authors along with their affiliated organizations, and country of origin. Each publication was then hand-searched to identify evidence level, keywords, and the methodology of the study. The missing data was then cross-matched with the ES database to ensure the accuracy and correctness of collected information.

### 2.4. Methodological Design

The publications were then categorized according to the methodology of the study as review articles, expert opinion, clinical practice guidelines, cross-sectional study, new material or technique, clinical studies, and laboratory studies.

### 2.5. Institution and Country of Origin

The author’s affiliation and origin country of publication were retrieved from the ES database as complete information for the marked list was not available from the WoS database. The retrieved information was then hand-searched and compared with the original text for each manuscript. Although corresponding addresses are considered a reliable source to identify the country of origin of publication; however, upon searching manually, it was seldom recorded. Each institution contributing to the publication was recorded as a single entry.

### 2.6. Data Analysis

The “Visualization of Similarities (VOS) viewer software” is widely used to graphically illustrate the bibliometric parameters in mapping networks, which allow easy visualization of critical elements [2,19,20,21]. The current study used VOS to represent a graphical mapping of keywords as identified bibliometric analysis to identify the focus of research in recent decades.

### 2.7. Statistical Analysis

The descriptive data and associations of citation frequency, citation density, publication age, and CCI were analyzed using IBM SPSS Statistics^®^, version 22, using the Spearman rank test. The normality of data was checked using the Shapiro–Wilk test. To explore the difference between two or more independent groups, the Kruskal–Wallis test was performed. Post-hoc testing was performed to confirm the difference between variables. Mann–Kendall trend test was performed to determine increasing and decreasing time trends. A *p*-value of < 0.05 was considered statistically significant.

## 3. Results

### 3.1. Bibliometric Parameters

**Figure 1:**
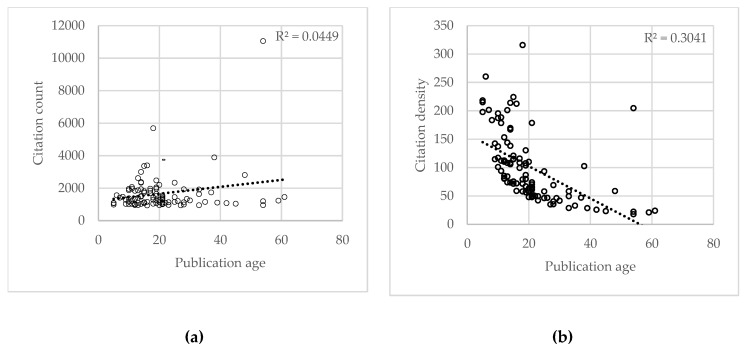
(**a**) Association of citation frequency with the age of publication (years). (**b**) Changes in trends of citation density with the age of publication.

The marked list of top 100 classics received a sum of 167,320 citations based on WoS, 165,947 citations based on ES, and 262,727 based on the GS database. The frequency of citations ranged from 940 to 11,051 (WoS), 1053 to 10,740 (ES), and 1162 to 20,041 (GS). Citation density is defined as the average number of citations/annum; it was calculated as 2742 (WoS), 2720 (ES), and 4307 (GS) for the 100 classics. “Antibiotic susceptibility testing by a standardized single disk method” was identified as the most cited “classic” with 11,051, 10,740, and 20,041 citations according to WoS, ES, and GS databases, respectively, with a citation density of 205 [22]. “Antimicrobial peptides of multicellular organisms” was ranked second with 5685, 5668, 7994 citations according to WoS, ES, and GS databases, respectively, with a citation density of 316 [23]. “Transformation of mammalian cells to antibiotic resistance with a bacterial gene under the control of the SV40 early region promoter” was ranked third with 3891, 2319, 3875 citations according to WoS, ES, and GS databases, respectively, with a citation density of 102 [24]. The marked list of top 100 classics along with their citation frequency from WoS, ES, and GS databases, publication age, citation density, and CCI 2019 is presented in Table 1. Shapiro–Wilk test revealed non-normal data on the citation frequency, citation density, and age of publication (years). Figure 1a shows a statistically significant upward trend of citation frequency was noted with the increase in publication age (R^2^ = 0.044, *p* = −0.012). Figure 1b shows a downward trend of citation density was noted with an increase in the age of publication (R^2^ = 0.304, *p* = −0.551), which was not statistically significant. The Supplementary Figure S1 illustrates the distribution of citation frequency over the last six decades.

### 3.2. Year of Publication

Chronologically, the oldest classic with 60 years of publication age was published in 1959 [60], and three articles with four years of publication age were published in 2015 [92,109,119] made it to the “classics” list. Fifty articles were published during 2000–2009, followed by 22 published during 1990–1999, 13 published during 2010–2019, seven published during 1980–1989, five published during 1959–1969, and three published during 1970–1979. Nine articles were published in 1999, marking it the year of most publications. Interestingly, 63% of the articles were published within the last two decades. The highest number of “classics” were published between 2000 and 2009 (*n* = 50).

### 3.3. Methodological Design and Evidence Level (EL) 

**Figure 2:**
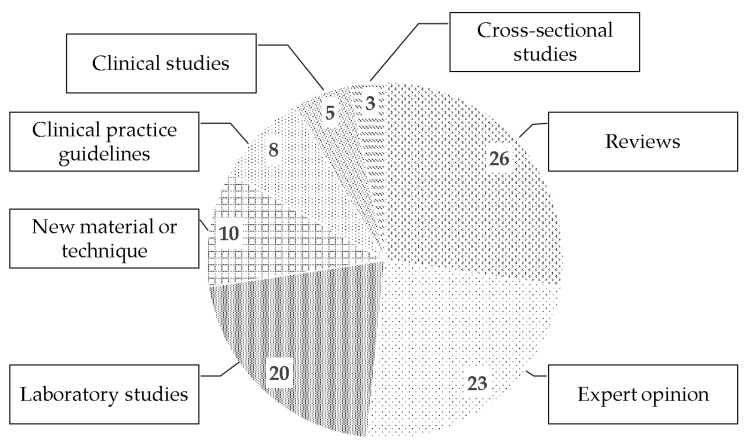
Pie chart diagram showing the distribution of classic articles based on the methodology of the study.

The distribution of the list based on methodological design is illustrated in Figure 2. Based on the level of evidence, 71 publications were graded as level-V, two were graded as level-IV, one belonged to level-III, four publications were graded as level-II, and 17 were graded as level-I. The evidence level and methodological design of five publications [24,52,60,77,111] were not identified as full-text of the articles were not accessible through different electronic sources. 

### 3.4. Contributing Authors, Institutions, and Countries

Robert E.W. Hancock was identified as the most contributing, authoring six classics, followed by Tomas Ganz, who contributed in four classics. A total of 513 authors contributed to the top 100 classics, among them 26 authors were contributed in two “classics” each. Complete texts for 95 publications were obtained, and five publications were not accessible through different institutions [24,52,60,77,111]. Based on the institutional address of the corresponding author as retrieved from the ES database, individuals from 26 countries contributed to the “classic” articles. Among these, 69 scientific contributions were from the United States of America. Followed by 18 publications from Canada, 11 from Germany, and four from Sweden. Three publications originated from Belgium, China, and Israel. Two publications originated from Egypt, Denmark, and India. One publication originated from, Argentina, Croatia, Ecuador, France, Kenya, Korea, Netherlands, New Zealand, Pakistan, South Africa, South Korea, Spain, Tanzania, Thailand, United Kingdom, and Australia.

Among 246 international institutions, the greatest contribution to the “classic” articles was made by the University of Manitoba, Canada, in six classics followed by the Stanford University School of Medicine, USA, in five classics. “University of Washington, USA”, “University of British Colombia, Canada”, “The University of California at Los Angeles, USA”, and “Harvard University, USA” contributed in four classics. “University of Kiel, Germany” and “University of California at San Diego, USA” contributed in three classics. “Robert Wood Johnson Medical School, USA”, “Weizmann Institute of Science, Israel”, “Emory University, Atlanta, Georgia, USA”, “Laurentian University, Ontario, Canada”, “Rush-Presbyterian-St. Luke’s Medical Center, USA”, “St. Agnes Medical Center, USA”, “the Centers for Disease Control and Prevention, Atlanta, Georgia, USA”, and “Veterans Affairs Palo Alto Health Care System, California, USA” contributed to two classics each.

### 3.5. Journal of Publication

**Figure 3:**
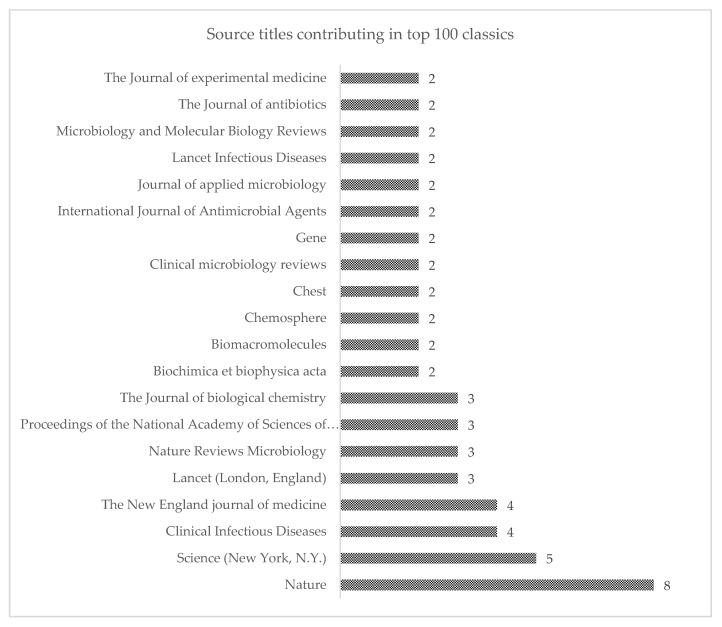
Bar graph representation of the number of articles published in different journals

The 100 classics were published across 63 different journals. Figure 3 presents the list of journals in which the highest number of classics were published. The list of the remaining journals is available as Supplementary Table S1.

### 3.6. Keywords

**Figure 4:**
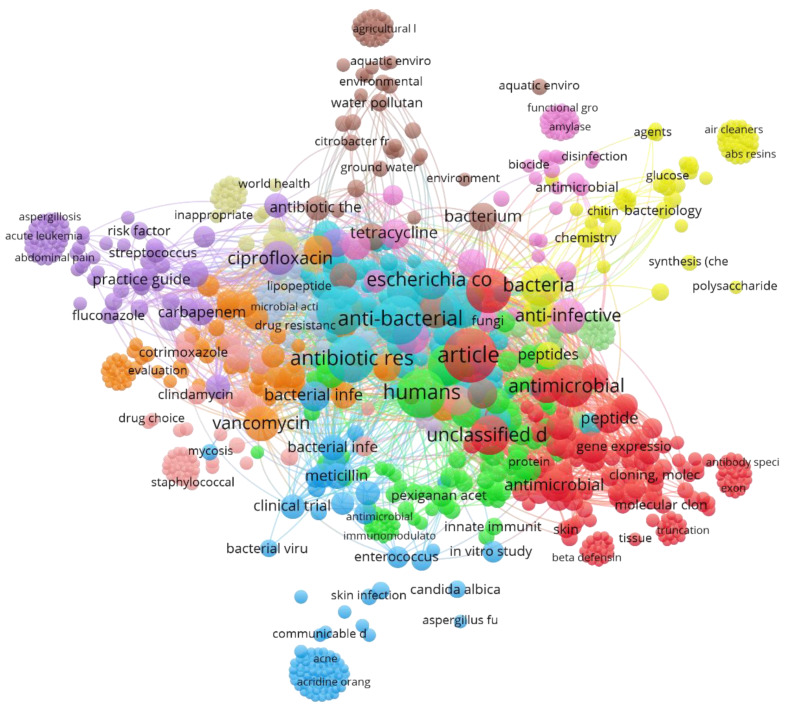
Network analysis of keywords identified from top 100 classics of antibiotics

The most frequently occurring keywords in the top 100 classics were “anti-bacterial agents” and “antibiotic agent”, followed by “antibiotic resistance”, “anti-infective agent”, and “antimicrobial”. Figure 4 is a graphical presentation of keywords arranged in a network of clusters. Colorful nodes represent the linkage of specific keywords to each cluster. Table S2 enlists the total number of index keywords and their frequency of occurrence based on the Elsevier Scopus database.

## 4. Discussion

The current study identified and analyzed the top 100 classics on antibiotics, antimicrobials, or antibacterial agents. Identification of any scientific contribution and inclusion in classics warrants the excellence and acclaimed acknowledgment by the relevant field experts, researchers, and scientists [12]. Theoretically, a higher citation frequency of a publication indicates the quality of the research conducted as identified by the scientific community [122]. Identification is imperative to study whether the classics have elaborated or explored the understanding of a problem and/or provided a comprehensive approach towards its solution, or whether the publication introduced a research trend or provided an expert opinion/summary on a topic of interest. The results of this study present the research perspective in the field of antibiotics, antimicrobials, or antibacterial agents for the last six decades. Additionlly, it illustrates key trends of research as well as clinical practice [2,8]. 

The definition of “classics” largely depends on the research field/specialty to which the publication belongs. In some fields, 100 or more citations of a publication are considered enough to classify it as a “classic” [6]. In perspective, the article ranked as 100th in the current study received 940 citations in comparison with the article ranked as 1st in the field of physics research in Korea that received 302 citations [123] or with the article ranked as 1st in the dental caries research that received 2003 citations [19]. For the current study, the publications receiving more than 400 citations can be considered classics. However, these publications will not make it to the top 100 due to the immense availability of the highly cited publications. 

Web of Science was used as a benchmark database because it has citation metrics from 1945 to the present [124]. A significant variance was observed when the citation metrics were cross-matched with other databases. The Elsevier Scopus database reports the citations dated back to 1996, which is a severe flaw while figuring out the most-cited papers. In contrast, the Google Scholar database counts the citations based on published articles, books, conference proceedings, thesis/dissertations, technical reports, and preprints, which explains the higher citation counts reported in the current study [2]. 

The current study found a statistically significant correlation of the citation frequency with the age of publication, which is similar to the findings of a previous bibliometric analysis report [2]. Although there was an upward trend of citations received by the classics to the age of publication [125], the trend analysis of the influence of age of publication on the citation density revealed that certain topics after reaching maturity show a decrease in citation density. This change in trend can be also be noticed from the current citation index 2019. 

It has been reported that the actual impact of a publication can only be assessed at least two decades after it has been published [2,4,17]. Interestingly, this phenomenon has been observed in the current study as the most number of classics were published in 1999. However, it is noteworthy that with the changing trends of how published work is reviewed, the accessibility of literature has increased multifold, and research from around the world can be remotely reviewed without needing access to archives, libraries, and published paper journals. This debate is backed up by the current study, which observed that 63 classics were published during the last two decades. This finding indicates that in the current era of digital technology, classics might require lesser years to reach their maturity stage.

With the evolution of research, several guidelines have been introduced to fulfill the ever-growing need for organized reporting of observational studies [126], laboratory studies [127], clinical studies [128], or reviews [129]. These guidelines allow the scrutinization of scientific information and improve the quality and transparency of reports. Preferred Reporting Items for Systematic Reviews and Meta-Analyses (PRISMA) statement is used to report systematic review and meta-analysis mainly focusing on evaluating randomized trials to provide the highest level of evidence. Surprisingly, the current study did not identify any systematic review of literature or meta-analysis, which made it to the list. The title of the study report is another key element which is stressed upon in various guidelines. It is entirely possible that some classics were not identified in the current study owing to how their titles were designed. A title should explicitly describe the methodology of study and key elements which identify the study to allow proper indexing of the article. 

Keywords play an essential role in the discoverability of any published article [130]. While searching any specific type of literature, scholars tend to methodically utilize search terms which are generally used in a specific field [131]. In this study, prime examples of such terms are antibiotics, antibacterials, or antimicrobials. However, it was noted that keywords only appeared in articles published after 1995 and more so not mandatorily in every publication. It was noted that even though keywords might have been submitted in the journal database during submission of manuscripts, the published articles did not display the keywords [55,63,109]. These incoherencies make the network analysis of keywords somewhat misleading and inconsistent with the actual data if we only rely on hand-searching. Therefore, the ES database was utilized to retrieve the relevant data to allow a presentable and fair network analysis.

## 5. Limitations

Firstly, a large amount of “classic” articles had to be excluded from the list as it was not considered possible to perform the bibliometric analysis of 500 or more articles in the current study. Therefore, the top 100 classics which achieved the maximum citations were selected for the present study. Secondly, the most recently published research papers are at a disadvantage irrespective of their content and quality, since they were outside the time window considered. Under this spectrum, it would not be wrong to say that the real impact of a research article cannot be accurately determined for at least five years post-publication. 

## 6. Conclusions

This bibliometric analysis of the top 100 classics on antibiotics revealed that the increase in the age of publication positively influenced the citation frequency. Unlike times before 1996, the explosion of access to scientific articles in the current era of digital technology means that classics written more recently might require fewer years to reach their mature stage. In spite of substantial developments and advancements in this field/specialty in recent decades, there is a dearth of systematic reviews and meta-analyses among the top 100 publications. Keywords are the cornerstones of the discoverability of any manuscript and therefore, quality journals and publishers should mandate the inclusion of keywords in every publication to ensure maximum visibility of the publication across all databases.

## Figures and Tables

**Table 1:** List of 100 classics of antibiotics ranked based on their citation frequency according to the Web of Science, Scopus, and Scholar databases along with citation density and current citation index (2019).

R ^1^	Author [Reference]	Year	CD ^2^	CCI ^3^ 2019	WoS ^4^	ES ^5^	GS ^6^
1	Bauer, Kirby, Sherris, and Turck [22]	1966	205	621	11,051	10,740	20,041
2	Zasloff [23]	2002	316	398	5685	5668	7994
3	Southern and Berg [24]	1982	102	3	3891	2319	3875
4	Cowan [25]	1999	179	292	3749	4598	11203
5	Sondi and Salopek-Sondi [26]	2004	212	353	3397	3677	5471
6	Brogden [27]	2005	224	302	3363	3353	4941
7	Kumar et al. [28]	2006	214	291	2996	3185	5039
8	Cohen et al. [29]	1972	59	17	2809	1775	3754
9	Kim et al. [30]	2007	201	311	2615	2818	4164
10	Stewart and Costerton [31]	2001	130	217	2474	2602	4113
11	Hancock and Sahl [32]	2006	170	139	2373	2391	3185
12	Kovach et al. [33]	2006	167	244	2337	2319	3019
13	Liu et al. [34]	1995	93	166	2332	2458	3571
14	Dorman and Deans [35]	2000	110	177	2201	2407	4479
15	Sharma et al. [36]	2009	188	240	2071	2269	3196
16	Mah and O’Toole [37]	2001	108	183	2043	2127	3529
17	Neu [38]	2003	116	119	1970	2071	3413
18	Chopra and Roberts [39]	2001	104	230	1967	2026	3414
19	Davies and Davies [40]	2009	178	312	1963	2037	3817
20	Ganz [41]	2010	195	395	1952	1983	3115
21	Zasloff [42]	2006	138	257	1935	1805	2643
22	Kuemmerer [43]	1992	69	82	1930	2007	2734
23	Dellit et al. [44]	1987	58	67	1915	1951	1732
24	Wiegand et al. [45]	2010	187	146	1871	1898	2848
25	Yeaman and Yount [46]	2007	144	146	1869	1846	2702
26	Nathan et al. [47]	2003	108	169	1842	1246	2033
27	Cushnie and Lamb [48]	2008	153	384	1839	2052	3983
28	Goossens et al. [49]	2005	121	230	1811	1850	2904
29	Sarmah et al. [50]	1983	47	31	1737	1817	2638
30	Kumarasamy et al. [51]	2005	115	149	1726	1842	3071
31	Mast et al. [52]	2005	115	70	1719	645	1868
32	Rabea et al. [53]	2003	99	171	1689	1719	2523
33	Anthonisen et al. [54]	2001	87	38	1650	1898	3065
34	Magill et al. [55]	1987	49	57	1633	1601	2294
35	Niederman et al. [56]	2014	260	337	1562	1893	2319
36	Liang et al. [57]	1999	74	96	1552	1525	2168
37	Zankari et al. [58]	2001	80	81	1518	1489	2039
38	Gewirtz [59]	2006	108	102	1512	1550	2177
39	Steers et al. [60]	2006	106	121	1482	596	1288
40	Hirsch et al. [61]	1999	70	82	1474	1549	2469
41	Jenssen et al. [62]	2012	184	484	1468	1462	2257
42	Laxminarayan et al. [63]	1959	24	1	1453	1454	2387
43	Park et al. [64]	1995	58	30	1447	1511	2483
44	Kohanski et al. [65]	2002	79	22	1421	1431	2062
45	Shai [66]	2013	201	326	1410	1403	1990
46	Boman [67]	2007	108	147	1405	1420	2100
47	Hoiby et al. [68]	1999	66	65	1391	1395	2249
48	Dethlefsen et al. [69]	2010	137	201	1372	1373	1981
49	Hughes et al. [70]	2008	112	124	1347	1641	1790
50	Nathan and Hibbs [71]	1999	64	99	1346	1230	1789
51	Li et al. [72]	1991	46	25	1336	1399	1975
52	Hidron et al. [73]	2008	111	72	1334	1418	2028
53	Hammer et al. [74]	2008	110	146	1323	1492	3055
54	Ong et al. [75]	2002	71	70	1286	1446	2058
55	Herrero et al. [76]	2011	142	165	1280	1205	1714
56	Burke [77]	1999	61	45	1278	1007	1773
57	Kollef et al. [78]	2001	67	45	1269	1462	2254
58	Freifeld et al. [79]	2000	63	45	1259	1493	2753
59	Ibrahim et al. [80]	1990	41	46	1243	1405	2098
60	Pigeon et al. [81]	1961	21	18	1234	1268	1917
61	Bennett et al. [82]	2009	112	149	1232	704	1187
62	Chambers and DeLeo [83]	1994	47	29	1213	1209	2058
63	Davies [84]	1966	22	4	1194	1294	2295
64	Cherepanov and Wackernagel [85]	2010	117	184	1171	1157	1708
65	Kong et al. [86]	1995	46	79	1154	1233	1741
66	Hamblin and Hasan [87]	2000	58	33	1153	1226	1740
67	Carter et al. [88]	1985	33	34	1152	1155	1677
68	Ganz et al. [89]	2004	72	112	1152	1024	1628
69	Ceri et al. [90]	1997	49	17	1135	1159	1716
70	Classen et al. [91]	2005	75	46	1129	1318	2194
71	Ventola [92]	1992	40	34	1128	1214	2398
72	Baddour et al. [93]	1999	53	98	1119	1198	1889
73	Bartlett et al. [94]	1981	28	45	1110	949	1625
74	Lande et al. [95]	1998	50	35	1099	1119	1597
75	Harder et al. [96]	2007	84	84	1096	1128	1716
76	Hancock and Lehrer [97]	2015	218	458	1091	1055	1628
77	Shai [98]	1978	26	21	1080	1074	1500
78	Rothstein et al. [99]	2015	215	413	1075	1087	1402
79	Steiner et al. [100]	2001	56	38	1072	968	1615
80	Ruparelia et al. [101]	2005	71	58	1071	1080	1518
81	Dethlefsen and Relman [102]	1975	23	65	1045	1045	1533
82	Fischbach and Walsh [103]	1999	50	51	1044	1031	1603
83	Vezina et al. [104]	2002	58	70	1041	1118	1699
84	Hancock and Chapple [105]	2009	94	99	1034	1058	1608
85	Andersson and Hughes [106]	2011	115	161	1032	1021	1625
86	Harder et al. [107]	2006	74	144	1029	1069	1684
87	Epand and Vogel [108]	2008	85	135	1022	1009	1442
88	Ling et al. [109]	2010	101	154	1009	1010	1594
89	Cohen [110]	1999	48	35	1004	1067	1857
90	Umezawa et al. [111]	1992	35	23	990	814	1214
91	Cabello [112]	2015	198	198	989	1049	1691
92	Kenawy et al. [113]	2008	81	135	975	1000	1303
93	Hancock [114]	1997	42	30	968	991	1448
94	Moazed and Noller [115]	2008	80	84	963	900	1281
95	Baquero et al. [116]	2007	74	91	961	1015	1587
96	Spellberg et al. [117]	1966	18	17	959	988	1598
97	Wang et al. [118]	2000	48	46	956	985	1476
98	Zhang et al. [119]	1987	29	27	947	1053	1162
99	Krause et al. [120]	1993	35	30	944	950	1549
100	Prezant et al. [121]	2004	59	59	940	906	1397

^1^ R = rank; ^2^ C.D. = citation density;^3^ CCI = current citation index;^4^ WoS = Web of Science;^5^ ES = Elsevier Scopus;^6^ GS = Google Scholar.

## Supplementary Materials

The following are available online at https://www.mdpi.com/2079-6382/9/5/219/s1, Figure S1: Distribution of citations frequency over last six decades, Table S1: List of journals which published top 100 classics, Table S2: List of keywords identified from the Elsevier Scopus database.
