# Supplementary material for: Antibiotics: A Bibliometric Analysis of Top 100 Classics
Source: Antibiotics (Basel). 2020 Apr 29;9(5):219. doi: 10.3390/antibiotics9050219 (PMC7277750; doi:10.3390/antibiotics9050219)
Supplement: Supplementary file 1 [file antibiotics-09-00219-s001.zip › Table S1.docx]

| Journal title | No. of publication |
| --- | --- |
| Nature | 8 |
| Science (New York, N.Y.) | 5 |
| Clinical Infectious Diseases | 4 |
| The New England journal of medicine | 4 |
| Lancet (London, England) | 3 |
| Nature Reviews Microbiology | 3 |
| Proceedings of the National Academy of Sciences of the United States of America | 3 |
| The Journal of biological chemistry | 3 |
| Biochimica et biophysica acta | 2 |
| Biomacromolecules | 2 |
| Chemosphere | 2 |
| Chest | 2 |
| Clinical microbiology reviews | 2 |
| Gene | 2 |
| International Journal of Antimicrobial Agents | 2 |
| Journal of applied microbiology | 2 |
| Lancet Infectious Diseases | 2 |
| Microbiology and Molecular Biology Reviews | 2 |
| The Journal of antibiotics | 2 |
| The Journal of experimental medicine | 2 |
| Acta Biomaterialia | 1 |
| Advances in Colloid and Interface Science | 1 |
| American journal of clinical pathology | 1 |
| American journal of respiratory and critical care medicine | 1 |
| Annals of internal medicine | 1 |
| Annual review of immunology | 1 |
| Antibiotics & chemotherapy (Northfield, Ill.) | 1 |
| Antimicrobial agents and chemotherapy | 1 |
| Applied microbiology | 1 |
| Biochemical pharmacology | 1 |
| Biopolymers | 1 |
| Cell | 1 |
| Circulation | 1 |
| Critical care medicine | 1 |
| Current Opinion in Biotechnology | 1 |
| Current opinion in immunology | 1 |
| Environmental microbiology | 1 |
| Environmental Science & Technology | 1 |
| FEBS letters | 1 |
| Infection Control and Hospital Epidemiology | 1 |
| International Journal of Food Microbiology | 1 |
| Journal of Antimicrobial Chemotherapy | 1 |
| Journal of bacteriology | 1 |
| Journal of clinical microbiology | 1 |
| Journal of colloid and interface science | 1 |
| Journal of immunology (Baltimore, Md. : 1950) | 1 |
| Journal of molecular and applied genetics | 1 |
| MMWR. Recommendations and reports : Morbidity and mortality weekly report. Recommendations and reports | 1 |
| Nanomedicine-Nanotechnology Biology and Medicine | 1 |
| Nature biotechnology | 1 |
| Nature genetics | 1 |
| Nature Protocols | 1 |
| Nature reviews. Immunology | 1 |
| P & T : a peer-reviewed journal for formulary management | 1 |
| Pharmacological reviews | 1 |
| Photochemical & photobiological sciences : Official journal of the European Photochemistry Association and the European | 1 |
| Plos Biology | 1 |
| Surgery | 1 |
| The Journal of clinical investigation | 1 |
| The Science of the total environment | 1 |
| Trends in biotechnology | 1 |
| Trends in microbiology | 1 |
| Water Research | 1 |
