# Supplementary material for: Antibiotics: A Bibliometric Analysis of Top 100 Classics
Source: Antibiotics (Basel). 2020 Apr 29;9(5):219. doi: 10.3390/antibiotics9050219 (PMC7277750; doi:10.3390/antibiotics9050219)
Supplement: Supplementary file 1 [file antibiotics-09-00219-s001.zip › Table S2.docx]

| Keywords | *Occurrences* |
| --- | --- |
| anti-bacterial agents | *39* |
| antibiotic agent | *39* |
| antibiotic resistance | *35* |
| antiinfective agent | *32* |
| bacteria (microorganisms) | *28* |
| antimicrobial activity | *27* |
| animals | *26* |
| bacteria | *25* |
| escherichia coli | *24* |
| unclassified drug | *23* |
| antibiotics | *19* |
| anti-infective agents | *18* |
| staphylococcus aureus | *18* |
| vancomycin | *18* |
| ciprofloxacin | *16* |
| controlled study | *16* |
| drug resistance, microbial | *15* |
| antimicrobial cationic peptides | *14* |
| molecular sequence data | *14* |
| animalia | *13* |
| amino acid sequence | *12* |
| antibacterial activity | *12* |
| bacterial infections | *12* |
| microbial sensitivity tests | *12* |
| polypeptide antibiotic agent | *12* |
| tetracycline | *12* |
| ampicillin | *11* |
| bacterium | *11* |
| gentamicin | *11* |
| microbiology | *11* |
| peptide | *11* |
| pseudomonas aeruginosa | *11* |
| unindexed drug | *11* |
| antibiotic sensitivity | *10* |
| drug effect | *10* |
| drug resistance, bacterial | *10* |
| erythromycin | *10* |
| female | *10* |
| male | *10* |
| quinoline derived antiinfective agent | *10* |
| sulfonamide | *10* |
| adult | *9* |
| animal | *9* |
| bacterial infection | *9* |
| beta lactam antibiotic | *9* |
| carbapenem | *9* |
| drug mechanism | *9* |
| gene expression | *9* |
| major clinical study | *9* |
| meticillin | *9* |
| minimum inhibitory concentration | *9* |
| peptides | *9* |
| tobramycin | *9* |
| antibiotic therapy | *8* |
| bacterial growth | *8* |
| chloramphenicol | *8* |
| clinical trial | *8* |
| drug efficacy | *8* |
| gram positive bacterium | *8* |
| klebsiella pneumoniae | *8* |
| methicillin resistant staphylococcus aureus | *8* |
| mice | *8* |
| penicillin g | *8* |
| proteins | *8* |
| trimethoprim | *8* |
| aminoglycoside antibiotic agent | *7* |
| base sequence | *7* |
| ceftazidime | *7* |
| cell membrane | *7* |
| doxycycline | *7* |
| drug isolation | *7* |
| drug structure | *7* |
| gram negative bacterium | *7* |
| human cell | *7* |
| macrolide | *7* |
| mouse | *7* |
| penicillin derivative | *7* |
| practice guideline | *7* |
| support, non-u.s. gov't | *7* |
| united states | *7* |
| aged | *6* |
| bactericidal activity | *6* |
| cephalosporin derivative | *6* |
| cotrimoxazole | *6* |
| cross infection | *6* |
| drug use | *6* |
| enterococcus | *6* |
| fungi | *6* |
| gene expression regulation | *6* |
| genetics | *6* |
| in vitro study | *6* |
| infection | *6* |
| kanamycin | *6* |
| messenger rna | *6* |
| pexiganan acetate | *6* |
| risk assessment | *6* |
| staphylococcus | *6* |
| streptomycin | *6* |
| support, u.s. gov't, p.h.s. | *6* |
| tetracycline derivative | *6* |
| amoxicillin | *5* |
| animal experiment | *5* |
| antifungal activity | *5* |
| biosynthesis | *5* |
| cathelicidin | *5* |
| cephalosporin | *5* |
| chemistry | *5* |
| clindamycin | *5* |
| cloning, molecular | *5* |
| colistin | *5* |
| defensin | *5* |
| drug design | *5* |
| drug resistance | *5* |
| hospital infection | *5* |
| host resistance | *5* |
| human tissue | *5* |
| immune response | *5* |
| infection control | *5* |
| isolation and purification | *5* |
| linezolid | *5* |
| membrane permeability | *5* |
| metabolism | *5* |
| models, molecular | *5* |
| molecular genetics | *5* |
| multidrug resistance | *5* |
| peptide derivative | *5* |
| plasmid | *5* |
| protein conformation | *5* |
| protein expression | *5* |
| rna | *5* |
| rna, messenger | *5* |
| spectinomycin | *5* |
| streptococcus pneumoniae | *5* |
| structure activity relation | *5* |
| agriculture | *4* |
| amikacin | *4* |
| aminoglycoside | *4* |
| antibiotic prophylaxis | *4* |
| antifungal agent | *4* |
| antimicrobial agents | *4* |
| antiviral activity | *4* |
| bacterial gene | *4* |
| bacterial strain | *4* |
| bacterial virulence | *4* |
| bacteriology | *4* |
| beta defensin 2 | *4* |
| biofilm | *4* |
| candida | *4* |
| candida albicans | *4* |
| cathelicidin antimicrobial peptide ll 37 | *4* |
| cell wall | *4* |
| chitosan | *4* |
| clarithromycin | *4* |
| cytotoxicity | *4* |
| daptomycin | *4* |
| defensins | *4* |
| dermaseptin | *4* |
| drug potency | *4* |
| enterobacteriaceae | *4* |
| enzyme inhibition | *4* |
| fluconazole | *4* |
| fusidic acid | *4* |
| genes | *4* |
| genetic engineering | *4* |
| immunity | *4* |
| immunity, natural | *4* |
| immunology | *4* |
| indolicidin | *4* |
| innate immunity | *4* |
| insect | *4* |
| magainin 2 | *4* |
| methicillin resistant staphylococcus aureus infection | *4* |
| methodology | *4* |
| microbial community | *4* |
| microbiological examination | *4* |
| microorganism | *4* |
| molecular cloning | *4* |
| mutation | *4* |
| nanoparticles | *4* |
| negibacteria | *4* |
| nucleotide sequence | *4* |
| oxacillin | *4* |
| physiology | *4* |
| piperacillin plus tazobactam | *4* |
| polymyxin b | *4* |
| posibacteria | *4* |
| protein structure | *4* |
| protozoa | *4* |
| psoriasis | *4* |
| rifampicin | *4* |
| risk factor | *4* |
| sequence homology | *4* |
| short survey | *4* |
| silver | *4* |
| skin | *4* |
| sulfamethoxazole | *4* |
| time factors | *4* |
| toxicity | *4* |
| transmission electron microscopy | *4* |
| water pollutants, chemical | *4* |
| aciclovir | *3* |
| adolescent | *3* |
| agar | *3* |
| amino acids | *3* |
| animal tissue | *3* |
| antimicrobial cationic peptide | *3* |
| antimicrobial therapy | *3* |
| antivirus agent | *3* |
| aquatic environments | *3* |
| azithromycin | *3* |
| aztreonam | *3* |
| bacillus subtilis | *3* |
| bacterium culture | *3* |
| beta defensin | *3* |
| beta lactamase | *3* |
| beta-defensins | *3* |
| biofilms | *3* |
| catheter infection | *3* |
| cecropin a | *3* |
| cefalotin | *3* |
| cefepime | *3* |
| cefotaxime | *3* |
| cell death | *3* |
| child | *3* |
| citrobacter freundii | *3* |
| cytoplasm | *3* |
| disease course | *3* |
| disease resistance | *3* |
| diseases | *3* |
| disulfide bond | *3* |
| dna | *3* |
| dna, bacterial | *3* |
| drug industry | *3* |
| drug sensitivity | *3* |
| drug synthesis | *3* |
| drug targeting | *3* |
| drug therapy | *3* |
| enterobacter | *3* |
| enterococcus faecium | *3* |
| environmental impact | *3* |
| essential oil | *3* |
| fosfomycin | *3* |
| gene cassette | *3* |
| gene induction | *3* |
| genes, bacterial | *3* |
| glucose | *3* |
| ground water | *3* |
| growth inhibition | *3* |
| growth rate | *3* |
| haemophilus influenzae | *3* |
| hydrophobicity | *3* |
| ib 367 | *3* |
| imipenem | *3* |
| impetigo | *3* |
| insecta | *3* |
| keratinocyte | *3* |
| levofloxacin | *3* |
| liver | *3* |
| medical society | *3* |
| membrane binding | *3* |
| meropenem | *3* |
| microorganisms | *3* |
| minocycline | *3* |
| molecular weight | *3* |
| monocyte | *3* |
| monotherapy | *3* |
| mortality | *3* |
| mycobacterium tuberculosis | *3* |
| mycosis | *3* |
| nafcillin | *3* |
| nanoparticle | *3* |
| nanostructured materials | *3* |
| neomycin | *3* |
| nisin | *3* |
| norfloxacin | *3* |
| oils, volatile | *3* |
| oxazolidinone derivative | *3* |
| permeability | *3* |
| phenotype | *3* |
| plant extract | *3* |
| plasmids | *3* |
| pneumonia | *3* |
| polymerase chain reaction | *3* |
| polysaccharide | *3* |
| postoperative infection | *3* |
| protegrin | *3* |
| protein | *3* |
| protein protein interaction | *3* |
| pseudomonas | *3* |
| recombinant dna | *3* |
| recombination, genetic | *3* |
| ribosome rna | *3* |
| rna, ribosomal | *3* |
| skin infection | *3* |
| soft tissue infection | *3* |
| staphylococcus epidermidis | *3* |
| structure-activity relationship | *3* |
| synthesis (chemical) | *3* |
| tachyplesin | *3* |
| thyme oil | *3* |
| tigecycline | *3* |
| tuberculosis | *3* |
| urinary tract infection | *3* |
| virus | *3* |
| waste water | *3* |
| waste water management | *3* |
| acinetobacter | *2* |
| acinetobacter baumannii | *2* |
| adverse drug reaction | *2* |
| agents | *2* |
| alpha defensin | *2* |
| amino acid | *2* |
| amoxicillin plus clavulanic acid | *2* |
| animal cell | *2* |
| animal model | *2* |
| antibacterial | *2* |
| antibiotics, aminoglycoside | *2* |
| aquaculture | *2* |
| aquatic environment | *2* |
| aspergillus fumigatus | *2* |
| bacitracin | *2* |
| bacterial cell wall | *2* |
| bacterial membrane | *2* |
| bacteriocin | *2* |
| bacterium isolate | *2* |
| biochemistry | *2* |
| biocide | *2* |
| biodegradation | *2* |
| biological membranes | *2* |
| biopolymers | *2* |
| biotechnology | *2* |
| buforin ii | *2* |
| calcitriol | *2* |
| cancer chemotherapy | *2* |
| candidiasis | *2* |
| case report | *2* |
| catalysis | *2* |
| catheter-related infections | *2* |
| cecropin | *2* |
| cefixime | *2* |
| ceftaroline | *2* |
| ceftriaxone | *2* |
| cell membrane permeability | *2* |
| cells, cultured | *2* |
| cellular immunity | *2* |
| cellulose | *2* |
| centers for disease control and prevention (u.s.) | *2* |
| channel gating | *2* |
| chemical structure | *2* |
| child, preschool | *2* |
| chitin | *2* |
| chitosan derivative | *2* |
| chromosome 19 | *2* |
| clinical article | *2* |
| clostridium difficile | *2* |
| colony count, microbial | *2* |
| combination chemotherapy | *2* |
| communicable diseases | *2* |
| complementary dna | *2* |
| correlation analysis | *2* |
| cost benefit analysis | *2* |
| coughing | *2* |
| crystal structure | *2* |
| culture media | *2* |
| culture medium | *2* |
| cystic fibrosis | *2* |
| cytokine production | *2* |
| cytology | *2* |
| data analysis | *2* |
| defensin 1 | *2* |
| defensin 2 | *2* |
| device | *2* |
| diabetic foot | *2* |
| dicloxacillin | *2* |
| dilution | *2* |
| disease outbreaks | *2* |
| disinfection | *2* |
| dna damage | *2* |
| dna repair | *2* |
| dna, complementary | *2* |
| drinking water | *2* |
| drosocin | *2* |
| drug choice | *2* |
| drug cost | *2* |
| drug determination | *2* |
| drug effects | *2* |
| drug half life | *2* |
| drug indication | *2* |
| drug inhibition | *2* |
| drug residues | *2* |
| drug resistance, multiple, bacterial | *2* |
| drug safety | *2* |
| drug screening | *2* |
| environment | *2* |
| environmental monitoring | *2* |
| enzyme activity | *2* |
| epidemic | *2* |
| epithelium cell | *2* |
| evaluation | *2* |
| evidence based medicine | *2* |
| fever | *2* |
| flavone derivative | *2* |
| flavonoid | *2* |
| flavonol derivative | *2* |
| food preservation | *2* |
| fungus | *2* |
| gastrointestinal infection | *2* |
| gene function | *2* |
| gram negative infection | *2* |
| gram-negative bacteria | *2* |
| gramicidin s | *2* |
| helicobacter pylori | *2* |
| hepcidin | *2* |
| histatin | *2* |
| horizontal gene transfer | *2* |
| hospital | *2* |
| hospitalization | *2* |
| hospitals | *2* |
| host cell | *2* |
| human immunodeficiency virus | *2* |
| hydrogen peroxide | *2* |
| hydrolysis | *2* |
| hydrophilicity | *2* |
| hypotension | *2* |
| inappropriate prescribing | *2* |
| infant | *2* |
| infection resistance | *2* |
| inflammation | *2* |
| influenza | *2* |
| intensive care unit | *2* |
| interleukin 6 | *2* |
| isoniazid | *2* |
| keratinocytes | *2* |
| lactoferricin | *2* |
| lemon oil | *2* |
| length of stay | *2* |
| lincomycin | *2* |
| lincosamide derivative | *2* |
| lipopeptide | *2* |
| lipopolysaccharide | *2* |
| lipopolysaccharides | *2* |
| livestock | *2* |
| macrophage | *2* |
| macrophages | *2* |
| mammalia | *2* |
| mass spectrometry | *2* |
| mbi 226 | *2* |
| melittin | *2* |
| mersacidin | *2* |
| mice, transgenic | *2* |
| microbial viability | *2* |
| middle aged | *2* |
| molecular model | *2* |
| molecular structure | *2* |
| mucosa inflammation | *2* |
| murinae | *2* |
| nalidixic acid | *2* |
| nanostructures | *2* |
| nanotechnology | *2* |
| nephrotoxicity | *2* |
| neutropenia | *2* |
| nitric oxide | *2* |
| nucleic acid synthesis | *2* |
| ofloxacin | *2* |
| ototoxicity | *2* |
| oxidative stress | *2* |
| oxygen | *2* |
| oxytetracycline | *2* |
| patient safety | *2* |
| penicillin resistance | *2* |
| penicillins | *2* |
| piperacillin | *2* |
| plant | *2* |
| plant oils | *2* |
| plants (botany) | *2* |
| pneumonia, ventilator-associated | *2* |
| point mutation | *2* |
| polymer | *2* |
| polymerization | *2* |
| polymers | *2* |
| polysaccharides | *2* |
| pr 39 | *2* |
| preschool child | *2* |
| prescription | *2* |
| prevalence | *2* |
| prophylaxis | *2* |
| protein analysis | *2* |
| protein function | *2* |
| protein isolation | *2* |
| protein localization | *2* |
| pseudomonic acid | *2* |
| public health | *2* |
| pulsed field gel electrophoresis | *2* |
| quaternary ammonium derivative | *2* |
| quinolone | *2* |
| rats | *2* |
| regulatory mechanism | *2* |
| reproducibility | *2* |
| ribosome protein | *2* |
| rna translation | *2* |
| rna, bacterial | *2* |
| rna, ribosomal, 16s | *2* |
| saccharomyces cerevisiae | *2* |
| salmonella | *2* |
| scanning electron microscope | *2* |
| sediment | *2* |
| septic shock | *2* |
| sewage | *2* |
| silver nanoparticles | *2* |
| soil microbiology | *2* |
| staphylococcal infections | *2* |
| staphylococcal skin infection | *2* |
| stenotrophomonas maltophilia | *2* |
| streptococcus pyogenes | *2* |
| streptogramin | *2* |
| structure analysis | *2* |
| surface water | *2* |
| target cell | *2* |
| teichoic acid | *2* |
| telithromycin | *2* |
| temperature | *2* |
| textile industry | *2* |
| thorax radiography | *2* |
| thymus vulgaris | *2* |
| time | *2* |
| tissue | *2* |
| tissue distribution | *2* |
| titanium | *2* |
| titanium dioxide | *2* |
| toll like receptor | *2* |
| transformation, genetic | *2* |
| unspecified side effect | *2* |
| vancomycin resistant enterococcus | *2* |
| vertebrata | *2* |
| viruses | *2* |
| voriconazole | *2* |
| water | *2* |
| water contamination | *2* |
| water microbiology | *2* |
| water treatment | *2* |
| world health | *2* |
| wound infection | *2* |
| 1 (6 bromo 2 methoxy 3 quinolinyl) 4 dimethylamino 2 (1 naphthyl) 1 phenyl 2 butanol | *1* |
| abdominal pain | *1* |
| abs resins | *1* |
| abscess | *1* |
| ac 98 6446 | *1* |
| acne | *1* |
| acridine orange | *1* |
| acute leukemia | *1* |
| adenovirus | *1* |
| adjuvant therapy | *1* |
| ag colloids | *1* |
| agricultural land | *1* |
| air | *1* |
| air cleaners | *1* |
| air filters | *1* |
| air purification | *1* |
| alanine aminotransferase blood level | *1* |
| allogeneic hematopoietic stem cell transplantation | *1* |
| alpha hemolysin | *1* |
| alpha type phenol soluble modulin | *1* |
| amino terminal sequence | *1* |
| aminolevulinic acid | *1* |
| aminotransferase | *1* |
| amylase | *1* |
| analysis | *1* |
| animal cell culture | *1* |
| animal use | *1* |
| anti-infective agents, local | *1* |
| antibacterial properties | *1* |
| antibiotic biosynthesis | *1* |
| antibody specificity | *1* |
| antimicrobial activities | *1* |
| antimicrobial drugs | *1* |
| antimicrobial functional groups | *1* |
| antimicrobial polymers | *1* |
| antineoplastic agent | *1* |
| apramycin | *1* |
| arginine catabolic mobile element | *1* |
| aspartate aminotransferase blood level | *1* |
| aspergillosis | *1* |
| aspergillus | *1* |
| aspergillus niger | *1* |
| babesia divergens | *1* |
| babesiosis | *1* |
| bacillus anthracis | *1* |
| bacterial chromosome | *1* |
| bacterial colonization | *1* |
| bacterial disease | *1* |
| bacterial genome | *1* |
| bacterial resistance | *1* |
| bactericidal activities | *1* |
| bacterium carrier | *1* |
| bacterium mutant | *1* |
| bagg albino mouse | *1* |
| benzoporphyrin derivative | *1* |
| beta 2 microglobulin | *1* |
| beta defensin 3 | *1* |
| beta lactamase ampc | *1* |
| beta-defensin 3 | *1* |
| betaproteobacteria | *1* |
| bilirubin | *1* |
| biological organs | *1* |
| biological product | *1* |
| biological products | *1* |
| bithionol | *1* |
| bl 2060 | *1* |
| bladder disease | *1* |
| blood cell count | *1* |
| blood culture | *1* |
| bone infection | *1* |
| bpi 23 | *1* |
| brain abscess | *1* |
| burn infection | *1* |
| c reactive protein | *1* |
| capping agents | *1* |
| carbapenem derivative | *1* |
| carbonyl iron | *1* |
| carboxy terminal sequence | *1* |
| caspofungin | *1* |
| cefaclor | *1* |
| ceftobiprole | *1* |
| cefuroxime | *1* |
| cell culture | *1* |
| cell membranes | *1* |
| cell ultrastructure | *1* |
| cetrimide | *1* |
| chagas disease | *1* |
| chemical agents | *1* |
| chemical analysis | *1* |
| chemical bonds | *1* |
| chemical model | *1* |
| childhood disease | *1* |
| china | *1* |
| chlorine derivative | *1* |
| chromosome 7 | *1* |
| chymotrypsin | *1* |
| cilastatin plus imipenem | *1* |
| citrobacter | *1* |
| clavulanic acid | *1* |
| climate change | *1* |
| clinical practice | *1* |
| clinical trials | *1* |
| cloning | *1* |
| cls 001 | *1* |
| coagulase negative staphylococcus | *1* |
| colistimethate | *1* |
| colloid | *1* |
| colloid chemistry | *1* |
| colloids | *1* |
| common cold | *1* |
| communicable disease | *1* |
| communicable diseases, emerging | *1* |
| community associated methicillin resistant staphylococcus aureus | *1* |
| community-acquired infections | *1* |
| comparative study | *1* |
| comprehensive evaluation | *1* |
| concentration (composition) | *1* |
| conjugate | *1* |
| conventional methods | *1* |
| cosmetic | *1* |
| cost control | *1* |
| cost effectiveness | *1* |
| cost effectiveness analysis | *1* |
| cp 226 | *1* |
| cpg oligodeoxynucleotide | *1* |
| creatinine | *1* |
| creatinine blood level | *1* |
| csa 13 | *1* |
| cysteine | *1* |
| czen 002 | *1* |
| dalbavancin | *1* |
| decision support techniques | *1* |
| depsipeptide | *1* |
| depsipeptides | *1* |
| deuteroporphyrin derivative | *1* |
| developing country | *1* |
| disease model | *1* |
| disease models, animal | *1* |
| disease transmission | *1* |
| drug administration routes | *1* |
| drug delivery system | *1* |
| drug development | *1* |
| drug discovery | *1* |
| drug excretion | *1* |
| drug formulation | *1* |
| drug identification | *1* |
| drug products | *1* |
| drug protein binding | *1* |
| drug research | *1* |
| drug selectivity | *1* |
| drug stability | *1* |
| drug substitution | *1* |
| drug utilization | *1* |
| ear infection | *1* |
| ecological impact | *1* |
| eleftheria terrae | *1* |
| emission inventories | *1* |
| emission inventory | *1* |
| environmental concentration | *1* |
| environmental fate | *1* |
| environmental implications | *1* |
| environmental toxicities | *1* |
| environmentally friendly synthesis | *1* |
| enzyme degradation | *1* |
| epidemiology, molecular | *1* |
| epithelial cells | *1* |
| equine infectious anemia virus | *1* |
| ethylene vinyl acetate copolymer | *1* |
| etiopurpurin | *1* |
| evidence based practice | *1* |
| exon | *1* |
| experimental model | *1* |
| extended spectrum beta lactamase | *1* |
| feline leukemia virus | *1* |
| filtration | *1* |
| fluorescence | *1* |
| food contamination | *1* |
| food packaging | *1* |
| food quality | *1* |
| food safety | *1* |
| formularies, hospital | *1* |
| friend leukemia virus | *1* |
| fugacity | *1* |
| functional groups | *1* |
| functions of the skin and its appendages | *1* |
| gallidermin | *1* |
| gene | *1* |
| gene amplification | *1* |
| gene overexpression | *1* |
| genotype | *1* |
| glycylcycline derivative | *1* |
| gonorrhea | *1* |
| gram positive infection | *1* |
| green fluorescent protein | *1* |
| green synthesis | *1* |
| haemophilus influenzae type b | *1* |
| hb 107 | *1* |
| hb 50 | *1* |
| health auxiliary | *1* |
| health care delivery | *1* |
| health care personnel | *1* |
| health care quality | *1* |
| health impact | *1* |
| health practitioner | *1* |
| health program | *1* |
| health services needs and demand | *1* |
| helicobacter | *1* |
| hematoporphyrin derivative | *1* |
| hepc gene | *1* |
| hepcidin 20 | *1* |
| hepcidin 25 | *1* |
| herpes simplex | *1* |
| herpes simplex virus | *1* |
| herpes simplex virus 1 | *1* |
| herpes virus | *1* |
| hexadecyltrimethylammonium bromides | *1* |
| hexapoda | *1* |
| high risk patient | *1* |
| histidine | *1* |
| hlf 1 11 | *1* |
| hospital sector | *1* |
| host pathogen interaction | *1* |
| human activities | *1* |
| human t cell leukemia virus | *1* |
| hybrid protein | *1* |
| hybridization | *1* |
| hypericin | *1* |
| illumination | *1* |
| immunomodulation | *1* |
| immunomodulatory activities | *1* |
| immunoregulation | *1* |
| immunotoxicity | *1* |
| imx 942 | *1* |
| in plants | *1* |
| in vivo study | *1* |
| infection prevention | *1* |
| infection rate | *1* |
| influenza vaccination | *1* |
| interleukin 8 | *1* |
| intracellular localization | *1* |
| intron | *1* |
| iron | *1* |
| iron dextran | *1* |
| iron overload | *1* |
| irradiation | *1* |
| itraconazole | *1* |
| keratitis | *1* |
| klebsiella | *1* |
| kluyveromyces | *1* |
| knockout mouse | *1* |
| laboratory test | *1* |
| large scale production | *1* |
| leukemia | *1* |
| leukocyte count | *1* |
| level iii fugacity models | *1* |
| light | *1* |
| liver disease | *1* |
| lutetium texaphyrin | *1* |
| malachite green | *1* |
| malaria | *1* |
| medical applications | *1* |
| medical audit | *1* |
| medical education | *1* |
| medical practice | *1* |
| medical problems | *1* |
| metal nanoparticle | *1* |
| metal nanoparticles | *1* |
| metal refining | *1* |
| methicillin resistance | *1* |
| methicillin-resistant staphylococcus aureus | *1* |
| methylene blue | *1* |
| metronidazole | *1* |
| micafungin | *1* |
| mice, inbred balb c | *1* |
| microbial activity | *1* |
| microbial contamination | *1* |
| microbial sensitivity test | *1* |
| models, chemical | *1* |
| models, theoretical | *1* |
| molecular epidemiology | *1* |
| money | *1* |
| morpho-logies | *1* |
| mouth infection | *1* |
| moxifloxacin | *1* |
| multigene family | *1* |
| multilocus sequence typing | *1* |
| multimedia | *1* |
| mx 226 | *1* |
| myelodysplastic syndrome | *1* |
| myeloid leukemia | *1* |
| n (5 fluoro 1 oxido 2 pyridinyl) 1 [2 (n formyl n hydroxyaminomethyl)hexanoyl]prolinamide | *1* |
| national consumption | *1* |
| neoplasm | *1* |
| neutrophil count | *1* |
| newborn infection | *1* |
| niche | *1* |
| northern blotting | *1* |
| nose infection | *1* |
| nuclear localization signal | *1* |
| nuclear propulsion | *1* |
| nutritional status | *1* |
| omiganan | *1* |
| oral mucosal disease | *1* |
| organ specificity | *1* |
| organ transplantation | *1* |
| oritavancin | *1* |
| outer membrane | *1* |
| pac 113 | *1* |
| palladium bacteriopheophorbide | *1* |
| panton valentine leukocidin | *1* |
| parainfluenza virus infection | *1* |
| parasitosis | *1* |
| pathogen | *1* |
| pathogenicity | *1* |
| penicillin binding protein 2a | *1* |
| penicillinase | *1* |
| peptide synthesis | *1* |
| peptidoglycan | *1* |
| peptoclostridium difficile | *1* |
| pharmacist | *1* |
| pharmacy service, hospital | *1* |
| phosphonium derivative | *1* |
| photocatalytic activity | *1* |
| photochemotherapy | *1* |
| photodynamic therapy | *1* |
| photofrin | *1* |
| photosensitizing agent | *1* |
| photosensitizing agents | *1* |
| phototoxicity | *1* |
| phthalocyanine | *1* |
| physical examination | *1* |
| plasmodium falciparum | *1* |
| pollutant source | *1* |
| poly(methyl methacrylate) | *1* |
| polyacrylic acid | *1* |
| polyamide | *1* |
| polyethylene | *1* |
| polyethylene terephthalate | *1* |
| polylactic acid | *1* |
| polylysine | *1* |
| polymacon | *1* |
| polyoxometalate i | *1* |
| polyoxometallates | *1* |
| polyurethan | *1* |
| polyvinyl alcohol | *1* |
| polyvinylchloride | *1* |
| porphyrin derivative | *1* |
| posaconazole | *1* |
| practice guidelines | *1* |
| precursor | *1* |
| predicted environmental concentrations | *1* |
| prematurity | *1* |
| primary medical care | *1* |
| protein a | *1* |
| ptx 002 | *1* |
| ptx 005 | *1* |
| ptx 006 | *1* |
| ptx 007 | *1* |
| rat | *1* |
| rats, sprague-dawley | *1* |
| reaction kinetics | *1* |
| recombinant bactericidal permeability increasing protein | *1* |
| recycling | *1* |
| residual toxicity | *1* |
| respiratory syncytial pneumovirus | *1* |
| river | *1* |
| river basin | *1* |
| rivers | *1* |
| rose bengal | *1* |
| rural population | *1* |
| sedimentation | *1* |
| semliki forest alphavirus | *1* |
| sepsis | *1* |
| sequence homology, amino acid | *1* |
| sialidase inhibitor | *1* |
| silver colloid nanoparticles | *1* |
| silver compounds | *1* |
| silver derivative | *1* |
| simian immunodeficiency virus | *1* |
| single drug dose | *1* |
| skin physiology | *1* |
| small molecule libraries | *1* |
| societies, medical | *1* |
| software | *1* |
| soil microorganism | *1* |
| spatiotemporal analysis | *1* |
| sprague dawley rat | *1* |
| staphylococcal pneumonia | *1* |
| staphylococcus phage 3a | *1* |
| staphylococcus toxin | *1* |
| starch | *1* |
| streptococcus | *1* |
| streptococcus agalactiae | *1* |
| streptococcus sp. 'group b' | *1* |
| streptothricin | *1* |
| sugars | *1* |
| sulfamidochrysoidine | *1* |
| sultamicillin | *1* |
| sunscreen | *1* |
| surface active agents | *1* |
| surveys | *1* |
| synthesis | *1* |
| synthetic methods | *1* |
| synthetic procedures | *1* |
| teamwork | *1* |
| technology, pharmaceutical | *1* |
| teichoic acids | *1* |
| teixobactin | *1* |
| telavancin | *1* |
| tetrakis(3 hydroxyphenyl)chlorin | *1* |
| tetraphenylporphyrin derivative | *1* |
| textile finishing | *1* |
| theoretical model | *1* |
| therapeutic strategies | *1* |
| thiazine derivative | *1* |
| thrombocyte count | *1* |
| ticarcillin | *1* |
| tin derivative | *1* |
| tio2 | *1* |
| tio2 nanoparticles | *1* |
| tolonium chloride | *1* |
| toothpaste | *1* |
| total hip prosthesis | *1* |
| toxin analysis | *1* |
| triclosan | *1* |
| truncation | *1* |
| trypanosoma cruzi | *1* |
| tumor necrosis factor alpha | *1* |
| tungsten compounds | *1* |
| tungsten derivative | *1* |
| ultrastructural analyses | *1* |
| ultrastructure | *1* |
| uncultured bacterium | *1* |
| urban population | *1* |
| urea nitrogen blood level | *1* |
| urine level | *1* |
| vectors | *1* |
| vertebrates | *1* |
| vesicular stomatitis virus | *1* |
| virulence factor | *1* |
| virus infection | *1* |
| wastewater treatment | *1* |
| water management | *1* |
| water pollutant | *1* |
| water recycling | *1* |
| watersheds | *1* |
| wire | *1* |
| wound dressing | *1* |
| xanthene derivative | *1* |
| yeasts | *1* |
| zinc | *1* |
| zinc oxide | *1* |
