# Supplementary figures and images for: Antibiotics: A Bibliometric Analysis of Top 100 Classics
Source: Antibiotics (Basel). 2020 Apr 29;9(5):219. doi: 10.3390/antibiotics9050219 (PMC7277750; doi:10.3390/antibiotics9050219)

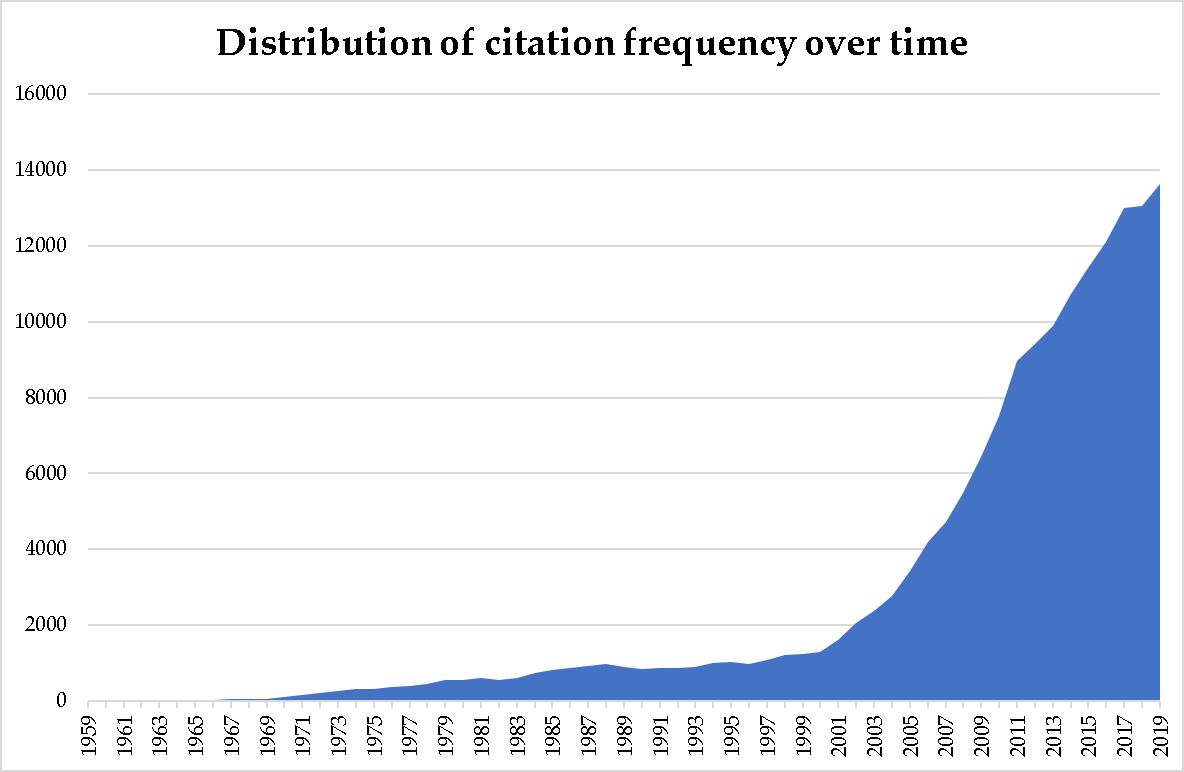

Supplement: Supplementary file 1 [file antibiotics-09-00219-s001.zip › Figure S1.tif]
